# EAT-Rice: A predictive model for flanking gene expression of T-DNA insertion activation-tagged rice mutants by machine learning approaches

**DOI:** 10.1371/journal.pcbi.1006942

**Published:** 2019-05-08

**Authors:** Chi-Chou Liao, Liang-Jwu Chen, Shuen-Fang Lo, Chi-Wei Chen, Yen-Wei Chu

**Affiliations:** 1 Institute of Molecular Biology, National Chung Hsing University, Taichung, Taiwan; 2 Advanced Plant Biotechnology Center National Chung Hsing University, Taichung, Taiwan; 3 Agricultural Biotechnology Center, National Chung Hsing University, Taichung, Taiwan; 4 Institute of Molecular Biology, Academia Sinica, Taipei, Taiwan; 5 Department of Computer Science and Engineering, National Chung Hsing University, Taichung, Taiwan; 6 Biotechnology Center, National Chung Hsing University, Taichung, Taiwan; 7 Ph.D. Program in Translational Medicine, National Chung Hsing University, Taichung, Taiwan; 8 Rong Hsing Research Center For Translational Medicine, National Chung Hsing University, Taichung, Taiwan; IIT Madras, INDIA

## Abstract

T-DNA activation-tagging technology is widely used to study rice gene functions. When T-DNA inserts into genome, the flanking gene expression may be altered using CaMV 35S enhancer, but the affected genes still need to be validated by biological experiment. We have developed the EAT-Rice platform to predict the flanking gene expression of T-DNA insertion site in rice mutants. The three kinds of DNA sequences including UPS1K, DISTANCE, and MIDDLE were retrieved to encode and build a forecast model of two-layer machine learning. In the first-layer models, the features nucleotide context (N-gram), cis-regulatory elements (Motif), nucleotide physicochemical properties (NPC), and CG-island (CGI) were used to build SVM models by analysing the concealed information embedded within the three kinds of sequences. Logistic regression was used to estimate the probability of gene activation which as feature-encoding weighting within first-layer model. In the second-layer models, the NaiveBayesUpdateable algorithm was used to integrate these first layer-models, and the system performance was 88.33% on 5-fold cross-validation, and 79.17% on independent-testing finally. In the three kinds of sequences, the model constructed by Middle had the best contribution to the system for identifying the activated genes. The EAT-Rice system provided better performance and gene expression prediction at further distances when compared to the TRIM database. An online server based on EAT-rice is available at http://predictor.nchu.edu.tw/EAT-Rice.

## Author summary

Among all the food crops, the rice is one of the staple foods in the human population, especially in Asia. However, the human population increases rapidly and the cultivated areas decrease in these decades. To solve the food crisis in the future, the rice researchers devote themselves to study on the gene function to increase the rice yield and stress tolerant ability. There are around 39000 annotated genes in rice, so scientists are hard to survey the gene functional because of the complexity and interactivity among the genes. Therefore, scientists put into a lot of manpower and funds into the field. The T-DNA (Transfer DNA) activation-tagging biotechnology has been wildly used on studies of rice gene function, however, it might influence the flanking genes expression when T-DNA inserted into the rice genome randomly. Thus, it will take lot of time for the researchers to validate the activation of genes by T-DNA enhancer. In these decades, as the increase of the biological data accumulation, the extraction of hidden information from this data is getting more and more important. To assist rice biologists in rapidly focusing the target gene affected by T-DNA. The application of machine learning methods in artificial intelligence (AI) and the establishment of prediction tool with biological data construction to correctly identify and classify target genes are of great significance in both theory and practice.

## Introduction

Rice is a major staple in the diet for more than half of the world’s human population. With the rapidly increasing pressures of both human population growth and global climate change, optimizing rice yields is critical over the next several decades. Sequencing of the rice genome, the smallest genome among the major cereal crops, was completed in 2005 [1] and from this work, rice emerged as the major monocot model plant for functional genome study and breeding improvement within cereal crops.

Global crop production, especially including maize, rice, wheat and soybean yields must double by 2050 to sustain the rapid growth of the World’s population [2]; therefore, rice scientists focus on intensive improvement of rice quality and yield as a primary goal, through the investigation of rice phenomics and genomics of which approximately 36500 genes have been annotated for application to functional genomics and modern breeding [3]. The International Rice Functional Genomics Project (IRFGP) has proposed an international coordinated project, RICE2020, to determine the biological function of every gene in the rice genome by 2020 [4]. Multiple methods for large-scale analysis of the biological function of genes by forward or reverse genetic approaches have been rapidly established, including bacterial artificial chromosome (BAC) libraries, large-scale expressed sequence tags (ESTs), full-length cDNA collections, a transcriptome database, transfer DNA (T-DNA) or transposon-tagged rice mutant populations, and genome-wide association study (GWAS)[5–15].

T-DNA insertional mutagenesis distributes uniformly throughout the rice genome, but preferentially in gene-rich regions, which results in knockout/loss-of-function for the inserted gene. Hence, this method may generate two questions that lead to fewer desirable plant traits: 1) Plant death occurs because the function of an essential gene is absent; 2) A disrupted gene can functionally complement via its gene family. To solve this problem, multiple tandem copies of cauliflower mosaic virus (CaMV) 35S enhancers [16] were introduced into a T-DNA vector for activation/gain-of-function tagging; genes within a 40–60 kb flanking region of the T-DNA-inserted locus are probably activated. Adding four 35S enhancer sequences in series to a T-DNA construction can enhance gene expression [16–21]. Development of large T-DNA mutant populations provides a powerful genetic resource for both forward and reverse genetics studies on gene function [5–8, 13, 14, 22].

The Taiwan Rice Insertional Mutant (TRIM) database was generated from Tainung 67 (TNG 67) and contains about 93,000 mutant lines; 85% and 65% of TRIM mutants have phenotyping and flanking sequence data, respectively [23], which significantly accelerates the ability to elucidate rice gene function. Three hundred genes of the flanking region of TRIM mutants were examined; 58% of these genes were activated by T-DNA insertion at differential levels [24] and demonstrating the activation of multiple activated genes became a laborious and time-consuming process.

Bioinformatics has developed rapidly [25, 26] and many biological prediction tools have been built by machine learning approaches [27–31]. Therefore, we developed a machine learning based tool for predicting the flanking gene expression around the T-DNA insertion site to assist researchers in improving the screening efficiency of activated genes.

We collected the validated genes by RT-PCR and clustered them into activated and non-detectable groups. DNA sequences including UPS1K (a 1 kb upstream sequence from the start codon), DISTANCE (from the start codon of a target gene to enhancer) and MIDDLE (a 150 bp up- and downstream sequence around the central nucleotide of the DISTANCE region) were retrieved to encode and build a two-layer machine learning prediction model. The features, containing N-gram, Motif, nucleotide physicochemical properties (NPC), and CG-island (CGI), were referenced to construct the first-layer models by support vector machines (SVM)[32]. Meanwhile, the logistic regression scoring, that take into account of the distance from target gene to T-DNA located site was used to weight the feature-encoding. In the second layer, because biological phenomena are caused by multiple factors, we analyzed different combinations of the four features noted above. In the second-layer models, the NaiveBayesUpdateable algorithm selected from 69 classified methods of the Waikato environment for knowledge analysis (WEKA) to integrate the first-layer models [33]. Our prediction platform, EAT-Rice, based on the TIGR MSU v7.0 genome, can predict genes within a specific range on both sides of the T-DNA insertion site and can provide a prediction outcome, confidence score, and the distance between T-DNA insertion site and target gene.

## Materials and methods

### Data sources and dataset preparation

For T-DNA activation-tagging, individual insertion events were confirmed by southern blot. Plasma rescue was used to find the T-DNA insertion site, then RT-PCR to detect the expression of genes around the T-DNA insertion site activated by enhancer. Two experimental datasets were collected: the first dataset included 226 T-DNA mutants containing 293 verified genes and the second dataset included 11 mutants containing 65 verified genes. Gene expression was divided into three types: activated gene (defined Ac), gene with no significant effect (defined NE), and non-detectable gene (defined ND)(Table 1). The first dataset of gene annotations were based on The Institute for Genomic Research Rice Genome Annotation project (TIGR)[34], and the second dataset was based on Rice Genome Automated Annotation System (RiceGAAS)[35]. Both of them in genome sequence were referenced from Oryza sativa japonica cv. Nipponbare. Each data in the dataset represents the target gene which was validated within its T-DNA mutant line; in other words, the same target gene in different mutant line was defined as the different data. Moreover, each data contained name of the mutant line, T-DNA insertion site, accession number, and the states of gene expression.

**Table 1:** Data distribution of flanking genes in rice T-DNA mutants.

Data Sources	Mutant Line	Gene Expression States			Validated Genes^a^
		Ac	NE	ND	
TDNA-DS1^b^	226	190	90	13	293
TDNA-DS2^c^	11	26	22	17	65
Sum	237	216	112	30	358

^a^ Validated gene indicated flanking gene expression of T-DNA mutants detected by RT-PCR.

^b^ TDNA-DS1 indicated the first collected dataset.

^c^ TDNA-DS2 indicated the second collected dataset.

Data for 30 non-detectable genes were collected but in order to ensure the quality and stability of our prediction system, these genes were removed. The no significant effect gene was defined as a non-activated gene (named NAc). The first dataset contained 280 genes, defined as the training set; the second dataset contained 48 genes, defined as the independent-testing set (Table 1). Two datasets come from different research units, which means that this data was made by different experimental process. We expect that the predictive model should have compatibility and practicality for the data from different research units; therefore, we applied TDNA-DS1 as training data and TDNA-DS2 as testing data rather than mixed the two datasets together. Thus, the method could also be used to validate the model whether it works in the study or not.

The ratio of positive data (indicated Ac) and negative data (indicated NAc) in training data may influence the efficiency of machine learning. First layer models of the training dataset with different proportions of positive and negative data were established. After evaluation, the optimal ratio of positive to negative data (P/N ratio) in 1:1 was obtained (S1 Fig). To divide the positive data into two section, we used the sequence similarity grouping. One sequence was selected within the population of 190 positive data compared with others using Pair-BLAST; the average of 189 scores was defined as the sequence similarity score. The flowchart for each positive data was duplicated to ensure all data were assigned a similarity score. Scores were sorted and divided into two groups (S2 Fig). To avoid losing data and optimal P/N ratio, 180 positive data was divided into two groups and merged 90 positive data in each group with the same negative data into training set of 180 data points named as training subset 1 and training subset 2, respectively.

### TRIM database

Taiwan Rice Insertional Mutant Database (TRIM, http://rice.sinica.edu.tw/fgb2/gbrowse/ TRIM_gb) which were built by Taiwan Academia Sinica can accelerate the rice functional research. The projects of TRIM are establishment of the mutant population, generation of genome-wide gene knockout by T-DNA, flanking sequence analysis, seed collection and phenotype characterization, seed conservation and PCR screening, inserted site in rice genome as well as the inserted orientation on the template are included. All above are to establish a database of the insertional mutant population. Biologists can survey whether the T-DNA mutants were inserted around the target gene which they are interested in because it might be suitable for gene functional study.

In this study, the T-DNA mutant lines are acquired from TRIM database, the expression levels of flanking genes were further identified. Our purpose is to effectively predict the effect of T-DNA insertions on flanking genes by the EAT-Rice, which will accelerate the research of Rice gene function by TRIM mutants.

### Sequence retrieving

**Fig 1:**
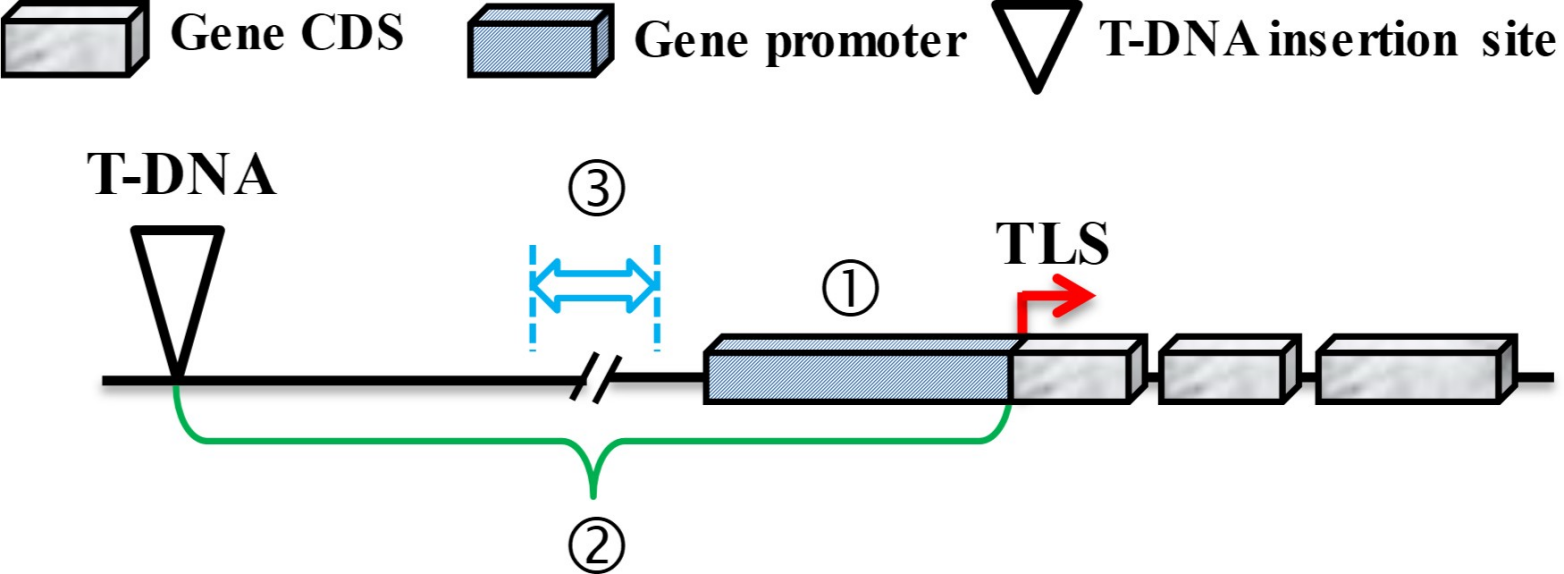
Illustration of three kinds of sequence information used in EAT-Rice construction. First region (slanted box) indicates UPS1K. Second region (curly bracket) indicates DISTANCE. Third region (double-headed arrow) indicates MIDDLE. The gene coding domain sequence (Gene CDS) of target gene is as grayish white box.

To analyze the difference in DNA sequences between activated (indicated Ac) and inactivated (NAc) genes, the three-part nucleotide sequence of the gene was retrieved, including UPS1K, DISTANCE, and MIDDLE. The three kinds of sequences retrieving followed the three hypotheses, which were supported in previous studies [36, 37]. 1) Based on promoter-enhancer interaction, first part of DNA fragment was one kb of upstream sequence from the start codon, also core promoter region, named as UPS1K; 2) In addition, based on scanning model, second part of DNA fragment was from the start codon of target gene to enhancer named as DISTANCE; 3) At last, based on lopping model, third part of DNA fragment was from 150 bp of up- and downstream sequence around the central nucleotide of DISTANCE region, and total length is 301 bp named as MIDDLE (Fig 1). T-DNA insertion site at upstream of target gene is an example shown as Fig 1. In fact, T-DNA may be inserted downstream of the target gene or intragenic. Therefore, the sequence length of the DISTANCE and MIDDLE will be changed depending on the T-DNA insertion site.

### Feature encoding

#### Nucleotide context (N-gram)

There were three points about the principle of N-gram. First, it chose specific sequences of DNA as template. Second, it searched the fragment of every nucleotide group to know frequency of occurrences of every fragment. Finally, it found representative fragments of sequences between Ac and NAc groups. These short fragments of nucleotide might be transcription factor biding site or motif. Three, four, five, and six-gram was applied to produce 64, 256, 1024, and 4096 types of nucleotide groups, respectively. Eq 1 was used to encode for N-gram models in different combinations (four types of gram coding), where *j* indicated encoding by frequency of occurrences of nucleotide fragments in specific area; total number of nucleotide groups was 5440.

$$NGRAM\_number_{\left(i\right)}=\left\{ \begin{array}{l} j,\;j\in N\\ 0,\;otherwise \end{array},\right.\;i\in \{1,2,\ldots ,5440\}$$ (1)

#### Regulatory cis-elements (Motif)

In the study, 2,087 verified motifs of regulatory cis-elements were collected [38] and the Find Individual Motif Occurrences (FIMO) tool within the Multiple Em for Motif Elicitation (MEME) suite was applied to search regulatory cis-elements on promoters [39]. The feature encoding was based on the result of the FIMO comparison. The 2,087 regulatory cis-elements were encoded based on number, conservation, orientation, density, and distance from a regulatory cis-element to translation start site (TLS) of the gene [40, 41], constructing a 12,522-dimensional training model.

Motif number is the frequency of motif occurrence within the promoter (*j*) represented in Eq 2. Motif conservation values were calculated using Eq 3 by adding all scores of a specific motif (M_*i*_), acquired by applying the FIMO tool, and dividing by the motif number. Motifs may be on the leading strand or lagging strand, therefore encoding was calculated by the ratio of motifs on positive and negative orientation. Positive orientation (specific motif on leading strand divide by motif number) was calculated using Eq 4; negative orientation (using the motif on the lagging strand) was calculated using Eq 5. Eq 6 was used to calculate motif density (indicating dispersed extent of a specific motif location on the promoter). Motif distance (distance between motif and T-DNA inserted site) was calculated using Eq 7; with multiple motif locations, each distance was calculated and summed, then divided by the motif number.

$${Motif\_Number}_{\left(i\right)}=\left\{ \begin{array}{l} \;j,\;j\in N\\ 0,otherwise \end{array}\right.,\;i\in \{1,2,\ldots ,2087\}$$ (2)

$${Motif\_Conserve}_{\left(i\right)}=\frac{M_{i}\;alignment\;score\;in\;promoter}{{Motif\_number}_{\left(i\right)}},i\in \{1,2,\ldots ,2087\}$$ (3)

$${Motif\_Pos.ori.}_{\left(i\right)}=\frac{pos\;in\;{Motif\_number}_{\left(i\right)}}{{Motif\_number}_{\left(i\right)}},i\in \{1,2,\ldots ,2087\}$$ (4)

$${Motif\_Neg.ori.}_{\left(i\right)}=\frac{neg\;in\;{Motif\_number}_{\left(i\right)}}{{Motif\_number}_{\left(i\right)}},i\in \{1,2,\ldots ,2087\}$$ (5)

$${Motif\_Density}_{\left(i\right)}=\frac{length\;of\;M_{i}\times {Motif\_number}_{\left(i\right)}}{distance\;of\;M_{i}\;distribution},i\in \{1,2,\ldots ,2087\}$$ (6)

$${Motif\_DisTG}_{\left(i\right)}=\frac{\mathrm{distance}\;\mathrm{from}\;T‐\mathrm{DNA}\;\mathrm{inserted}\;\mathrm{site}\;\mathrm{to}\;\mathrm{TLS}\;\mathrm{in}\;M_{i}}{{Motif\_number}_{\left(i\right)}},i\in \{1,2,\ldots ,2087\}$$ (7)

#### Nucleotide physicochemical and conformation properties (NPC)

The 125 types of dinucleotide physicochemical properties and structures from a dinucleotide properties database (DiProDB) (https://diprodb.leibniz-fli.de/) were integrated into 15 types by principal component analysis (PCA)[42]. A 240-dimensional training model using this feature was built to identify specificity of the target sequence (Eq 8).

$${NPC\_value}_{\left(i,j\right)}=\frac{S(d_{i})\times F_{j}(d_{i})}{sequence\;length-1},\;i\in \{1,2,\ldots ,16\},j\in \{1,2,\ldots ,16\},d_{i}\in D,F_{j}\in F,$$ (8)

where D is the combination of 16 types of dinucleotides for every property; F is 15 types of dinucleotide physicochemical properties; *S*(*di*) is frequency of occurrences of 16 dinucleotides on the target sequence; *Fj*(*di*) represents the value of 16 dinucleotides corresponding to each property in 15 dinucleotide physicochemical structures.

### CpG islands (CGI)

To determine the association of gene activation by analyzing whether CpG-island is present in promoter [43, 44]. The EMBOSS Newcpgreport tool from The European Bioinformatics Institute (EMBL-EBI) was used to predict CpG islands, and encoded by number, length, distance, CG ratio, and OE value (http://www.ebi.ac.uk/Tools/seqstats/emboss_newcpgreport/). CGI number was the number using Newcpgreport to predict CpG islands on the promoter of target gene (Eq 9). CGI length was value of the length of CG-island divided by the length of promoter (Eq 10). CGI distance was distance from CG-island to TLS of gene (Eq 11). The CG ratio of CGI was ratio of CpG dinucleotides in CG-island (Eq 12). The observed/expected (OE) value of CGI was ratio of number of CpG dinucleotides observed in CG-island to the expected number of CpG dinucleotides. Its formula was number of CpG dinucleotides on the promoter divided by number of cytosine nucleotide multiply number of guanine nucleotide on CpG-island (Eq 13).

$$CGI\_Number=\left\{ \begin{array}{l} j,\;j\in N\\ 0,\mathrm{otherwise} \end{array}\right.$$ (9)

$$CGI\_LengthRatio=\frac{length\;of\;CGI}{length\;of\;promoter}$$ (10)

CGI_Dis=|TLS−CGIlocation| (11)

$$CGI\_CGRatio=\frac{CpG\%\;in\;CGI}{CGI\_number}$$ (12)

$$CGI\_OE=\frac{number\;of\;CpG\;in\;CGI}{\left(number\;of\;C\;in\;CGI)\times (number\;of\;G\;in\;CGI\right)}$$ (13)

### Significant pattern selection between Ac and NAc genes

To reduce model complexity and shorten calculation time, we analyze the frequency of pattern occurrences of 5440 nucleotide groups of N-gram and 2087 regulatory cis-elements of Motif in the sequence, including UPS1K, DISTANCE, and MIDDLE, between Ac and NAc genes. DNA fragments with a P-value of < 0.05 by T-test (implemented by R) were selected to identify the patterns with different frequencies in the Ac and NAc sequences. For the N-gram, the UPS1K, DISTANCE, and MIDDLE, 359, 4085, and 349 patterns were filtered out with P-value < 0.05. In the Motif, 106 patterns were identified. The selected patterns above were encoded further depending on what the N-gram or motif it derived (An example was shown in S3 Fig).

### Model selected evaluation

In the research, a formula was designed to evaluate the prediction performance of the two second-layer models from training subset 1 and subset 2. We considered AUC, Sn, and Sp as our evaluating indicator in model, and the formula includes the value of cross-validation multiplied by the value of exchange-testing, divided by the value of self-consistency. Note, the formula indicates the lower the evaluating scores, the higher the extent of model overfitting, and vice versa.

$${Model(Eva)}_{i}=\frac{{Model}_{i}{Eva}_{cv}\times {Model}_{i}{Eva}_{Ex-test}}{{Model}_{i}{Eva}_{Self}},Eva\in \{AUC,Sn,Sp\}$$ (14)

$${Model}_{i}={Model(AUC)}_{i}+{Model(Sn)}_{i}+{Model(Sp)}_{i},where\;i=number\;of\;model$$

### Architecture of prediction system

**Fig 2:**
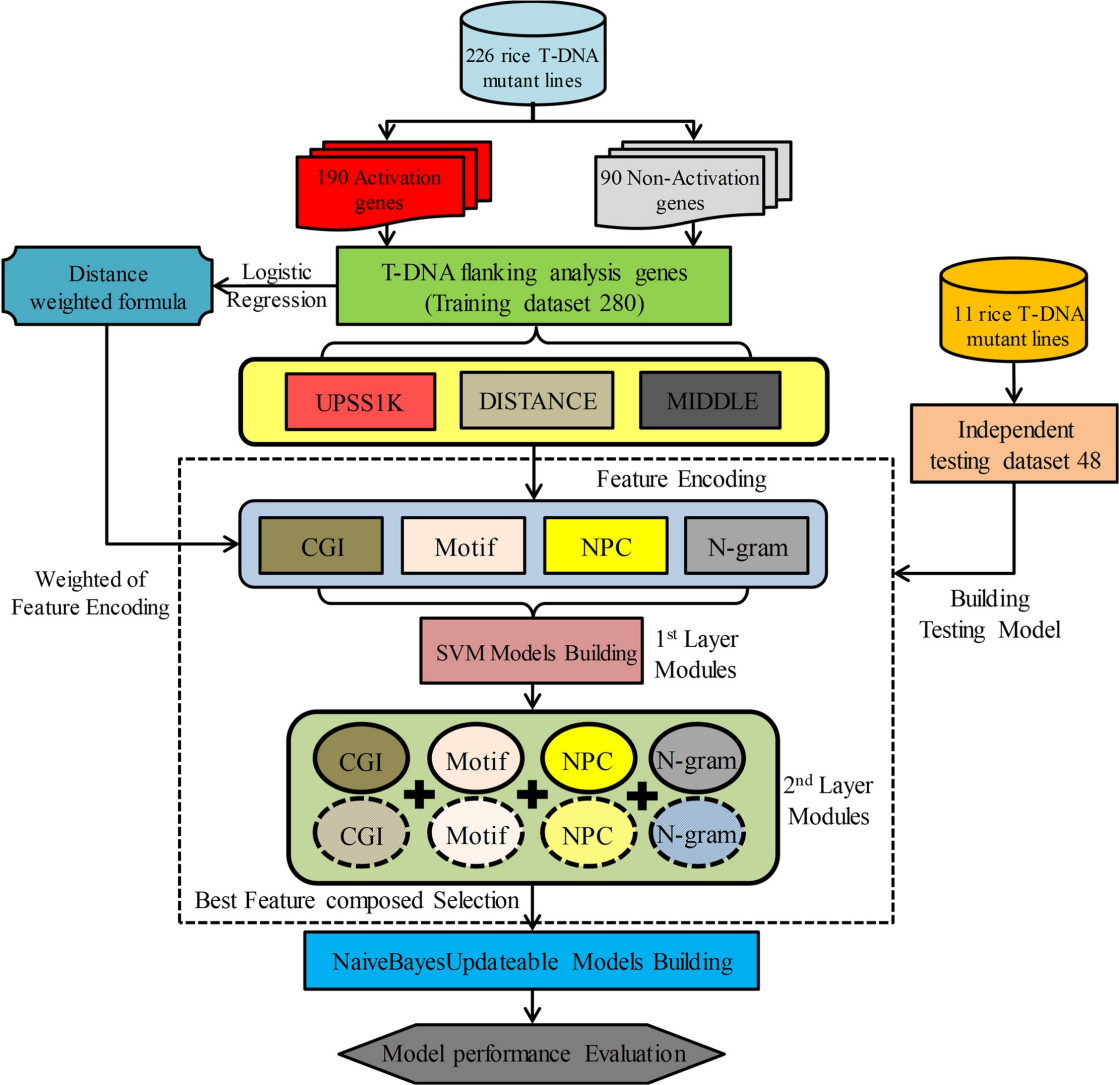
Flow chart of system architecture. The dotted line square indicates two-layer model construction. The solid and dotted circle line used for four kinds of features in 2^nd^ Layer Modules indicates feature combination mechanism.

In this study, we built the prediction system about the flanking gene expression of T-DNA insertion site in rice mutants by two layers model of machine learning. A 280 training set was selected to train a model of logistic regression based on the relationship between distance from the 35S enhancer to the target gene and gene expression. LIBSVM was used to build the first layer model that adopted three kinds of DNA sequences and four kinds of features for encoding. For UPS1K, four features, i.e., N-gram, Motif, NPC, and CGI, for encoding, while for DISTANCE and MIDDLE only N-gram and NPC were used to encode, and eight prediction models were generated (Table A in S1 Supplement). The optimal P/N ratio was calculated from the average results of eight models. For the second layer, we used a different combination to integrate first layer models encoded by four features, picked out the preferred model of predictive performance, and used WEKA v3.6 to analyze 69 kinds of classification algorithms. NaiveBayesUpdateable was adopted to build models (Table B in S1 Supplement). The accuracy of the two-layer model was evaluated with 48 independently testing data (Fig 2).

### Performance evaluation of model

A 5-fold cross-validation method and 48 verified genes were chosen as testing data to evaluate the predictive performance of the model; evaluation indictor were Accuracy (Acc), Sensitivity (Sn), Specificity (Sp), F-score (F1), and AUC (Area under the receiver operating characteristic curve). Acc can evaluate the prediction accuracy of positive and negative data; the closer to 100%, the more accurate the overall predictive performance of the model (Eq 15). Sn and Sp evaluate the accuracy of the prediction of positive and negative data, respectively (Eqs 16–17). F1 is the weighted average of Recall (also called Sn) and Precision (the ratio of true positive data with true positive data plus false positive data) of models (Eq 18). When the numbers of positive and negative data were different, Acc was not a good evaluation indicator, so we also considered AUC using an ROCR library of R language additionally. The Sn, Sp and AUC value are from 0 to 1. The closer to 1, the better learning of model.

$$Acc=\frac{TP+TN}{TP+FP+TN+FN}\times 100$$ (15)

$$Sn=\frac{TP}{TP+FN}$$ (16)

$$Sp=\frac{TN}{TN+FP}$$ (17)

$$F_{1}=2\times \frac{Precision\times Recall}{Precision+Recall}=\frac{2\times TP}{2\times TP+FN+FP}$$ (18)

## Results

### Relation between gene activation and distance from 35S enhancer

**Fig 3:**
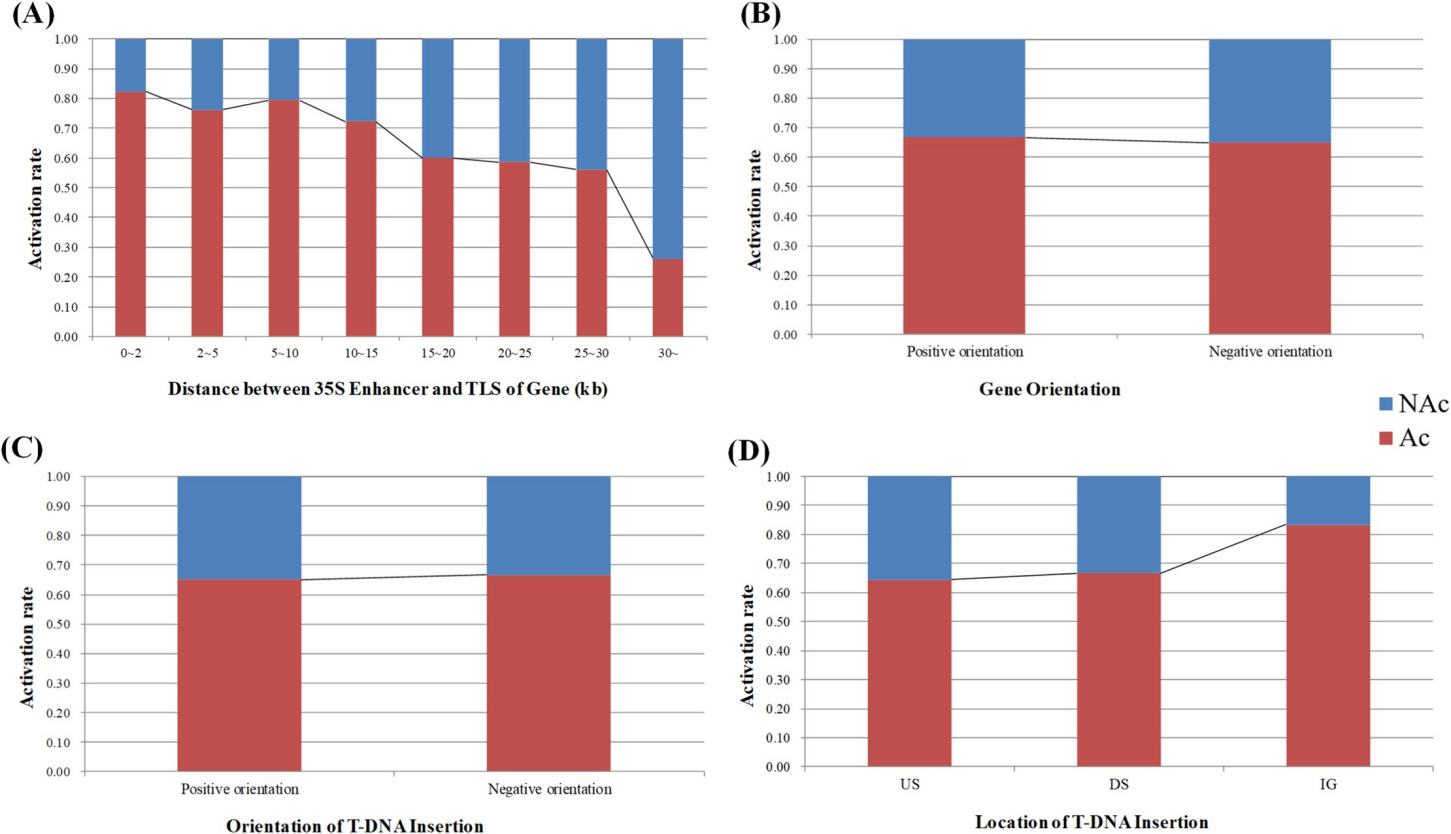
Correlation analysis of enhancer property and the activation ratio of genes. In the interaction between the enhancer and the target gene, we have summarized four properties including. (A) The distance from the 35S enhancer of the T-DNA insertion site to the TLS of gene. (B) Gene orientation. (C) Orientation of T-DNA insertion (enhancer's orientation). (D) Location of T-DNA insertion (enhancer’s location). US (Up-stream): T-DNA inserts into upstream of target gene, DS (Down-stream): T-DNA inserts into downstream of target gene, IG (Intragenic): T-DNA inserts into intragenic of target gene.

When we assigned the UPS1K sequence of the gene in the T-DNA activation-tagged mutant, we discovered 55 repeat sequences of different expression states, which are the result of a single target gene affected by multiple independent T-DNA insertion events. The data of these repeats differ significantly in the distance from the T-DNA insertion site to the target gene. We grouped data based on the distance from the 35S enhancer to the TLS of the gene and calculated the ratio of gene activation in detached groups that separated by distance. Statistical analysis showed that the distance between enhancer and TLS of the gene negatively correlated with gene activation (Fig 3A; Table C in S1 Supplement), implying that distance has the ability to influence the interaction between the enhancer and target gene.

Previous studies have suggested that the enhancer-gene interaction was not affected by orientation, location (i.e., the enhancer is located on the upstream, downstream or intragenic locus) and distance [17, 45]. However, our analysis demonstrated that there is a statistically significant difference in distance (P = 6.39e-07)(Fig 3A; Table D in S1 Supplement). Gene orientation, T-DNA insertion orientation, and location were analyzed to assess the promoter-enhancer interaction and if the probability of gene activation was influenced by these three factors. No significant effect for the three factors on the enhancer-to-gene activation was observed (Fig 3B–3D; Tables C and D in S1 Supplement).

The repeat sequences of different expression states may cause contradictions in model building by machine learning. Therefore, we used logistic regression to establish a model based on the distance factor to predict the probability of gene activation. The value of the regression prediction were used as a feature-encoding weighting when the first layer modules were built to distinguish repeat sequences, and the logistic regression formula was as shown in Eq 19:

$$\pi (x)=\frac{{exp}^{\left(1.448-7.099e-05x\right)}}{1+{exp}^{\left(1.448-7.099e-05x\right)}},$$ (19)

where linear regression formula is 1.448–7.099e-05*x*; intercept (fixed constant of linear regression) is 1.448; independent variable parameter is -7.099e-05; and *x* indicates distance variable. π(*x*) indicates the logically transformed function of the linear regression and represents the possibility of gene activation.

### Performance of the system with two-layer architecture in the subsets

The evaluation results on the first layer feature model of training subset 1 showed that the models constructed by UPS1K and MIDDLE in the N-gram encoding and UPS1K in Motif encoding achieved the most desirable results (Table 2). In models of UPS1K and MIDDLE using N-gram encoding, the cross-validation was 90.00% and 95.00% on Acc, while the independent-testing result for the same models was 64.58% and 72.92%, respectively. In the Motif model using N-gram encoding, training was 82.22% on Acc, but for the independent-testing of the Motif model using N-gram encoding, Acc was only 50.00%, indicating that this model may suffer from overfitting.

**Table 2:** Evaluation of the first layer of SVM feature model.

Feature Encoding	Sequence	Cross-Validation					Independent-Testing				
		*Acc(%)*	*AUC*	*F*_*1*_	*Sn*	*Sp*	*Acc(%)*	*AUC*	*F*_*1*_	*Sn*	*Sp*
N-gram	UPS1K	90.00	0.804	0.900	0.900	0.900	64.58	0.698	0.622	0.538	0.773
	DISTANCE	53.89	0.555	0.484	0.433	0.644	64.58	0.661	0.585	0.462	0.864
	MIDDLE	95.00	0.980	0.950	0.956	0.944	72.92	0.815	0.772	0.846	0.591
	Overall^a^	79.63	0.780	0.778	0.763	0.829	67.36	0.725	0.660	0.615	0.743
NPC	UPS1K	56.11	0.538	0.633	0.755	0.367	60.42	0.780	0.537	0.423	0.818
	DISTANCE	50.00	0.486	0.536	0.578	0.422	54.17	0.528	0.645	0.769	0.273
	MIDDLE	59.44	0.621	0.610	0.634	0.555	68.75	0.780	0.667	0.577	0.818
	Overall	55.18	0.548	0.593	0.656	0.448	61.11	0.696	0.616	0.590	0.636
Motif	UPS1K	82.22	0.879	0.826	0.844	0.800	50.00	0.490	0.571	0.615	0.364
CGI	UPS1K	51.67	0.526	0.62	0.789	0.245	50.00	0.439	0.613	0.731	0.227
Feature Encoding	Sequence	Cross-Validation					Independent-Testing				
		*Acc(%)*	*AUC*	*F*_*1*_	*Sn*	*Sp*	*Acc(%)*	*AUC*	*F*_*1*_	*Sn*	*Sp*
N-gram	UPS1K	81.11	0.888	0.811	0.811	0.811	70.83	0.638	0.759	0.846	0.545
	DISTANCE	61.67	0.613	0.615	0.611	0.622	58.33	0.743	0.444	0.308	0.909
	MIDDLE	89.44	0.940	0.897	0.922	0.867	70.83	0.823	0.781	0.962	0.410
	Overall	77.41	0.814	0.774	0.781	0.767	66.66	0.735	0.661	0.705	0.621
NPC	UPS1K	53.89	0.535	0.638	0.811	0.267	56.25	0.669	0.571	0.538	0.591
	DISTANCE	61.67	0.627	0.623	0.633	0.600	54.17	0.675	0.421	0.308	0.818
	MIDDLE	48.33	0.509	0.546	0.622	0.345	70.83	0.743	0.708	0.654	0.773
	Overall	54.63	0.557	0.602	0.689	0.404	60.42	0.696	0.567	0.500	0.727
Motif	UPS1K	79.44	0.844	0.798	0.811	0.778	64.58	0.661	0.691	0.731	0.545
CGI	UPS1K	49.44	0.471	0.480	0.466	0.522	41.67	0.484	0.588	0.769	0.000

^a^ Overall indicates average performance of models built by UPS1K, DISTANCE and MIDDLE sequence.

Model performance of training subset 2 was similar to subset 1. The range of expected model performance with 5-fold cross-validation was 79.44% - 89.44% and independent-testing was 64.58% - 70.83% on Acc. Compared with subset 1, the Motif model using N-gram encoding of subset 2 was >14.58% on Acc and was > 0.17 on AUC. In the N-gram encoding of subset 1 and subset 2, the DISTANCE model was 53.89% and 61.67% for cross-validation on Acc, respectively. However, for subset 1 and subset 2, the UPS1K model was approximately 36.11% and 19.44% greater than the DISTANCE model, and the MIDDLE model was also greater than 41.11% and 27.77%, respectively. In the NPC encoding, cross-validations of the models of UPS1K, DISTANCE, and MIDDLE in subset 1 and subset 2 averaged 55.18% and 54.63%, respectively; the average of independent-testing was 61.11% and 60.42%. In the CGI encoding, cross-validation and independent-testing were close to 50% on Acc, suggesting that CGI might not be a good classification feature. Taken together, N-gram and Motif classification performance was more valuable than NPC and CGI, indicating that some classification features have meaningful biological significance in this study.

To consider the complexity of the biological mechanism, the second layer models combined four features by integration of machine learning, with an eye to improving system accuracy. In the cross-validation of subset 1, we found that all evaluated parameters except the AUC demonstrated N-gram encoding provided a dominant contribution to classification (Table 3). The AUC value coincides with model performance; higher AUC value provide superior stability of the Ac and NAc gene classification in model performance. From these results, we selected the CGI+Motif+N-gram combination based on the highest AUC. The independent-testing results were Acc of 72.92%, AUC of 0.76, F1 of 0.772, Sn of 0.846, and Sp of 0.591. In the cross-validation of subset 2, the performance of a single N-gram was similar to that of subset 1, indicating that the contribution of N-gram in the second layer combination was more favorable. After considering the balance performance between AUC, Sn, and Sp, we selected two combinations of N-gram+NPC and CGI+N-gram+NPC. The results illustrated that both model performances were equivalent, implying that incorporation of the CGI feature did not improve accuracy. From this assessment, we selected the N-gram+NPC combination in subset 2. The independent-testing results were Acc of 79.17%, AUC of 0.806, F1 of 0.828, Sn of 0.923, and Sp of 0.636.

**Table 3:** Evaluation of the second layer of combination model using NaiveBayesUpdateable.

Pattern of Feature	Cross-Validation					Independent-Testing				
	*Acc(%)*	*AUC*	*F*_*1*_	*Sn*	*Sp*	*Acc(%)*	*AUC*	*F*_*1*_	*Sn*	*Sp*
N-gram	95.00	0.981	0.950	0.956	0.945	72.92	0.777	0.772	0.846	0.591
NPC	56.67	0.578	0.557	0.544	0.589	58.33	0.725	0.412	0.269	0.955
CGI	50.00	0.500	0.550	0.611	0.389	50.00	0.479	0.613	0.731	0.227
Motif	82.22	0.822	0.826	0.845	0.801	50.00	0.490	0.571	0.615	0.364
CGI+N-gram	95.00	0.982	0.950	0.956	0.945	72.92	0.783	0.772	0.846	0.591
CGI+NPC	50.56	0.561	0.508	0.511	0.500	58.33	0.734	0.412	0.269	0.955
CGI+Motif	82.22	0.822	0.826	0.845	0.801	50.00	0.484	0.571	0.615	0.364
N-gram+NPC	95.00	0.978	0.950	0.956	0.945	72.92	0.786	0.772	0.846	0.591
Motif+N-gram	94.45	0.987	0.944	0.945	0.945	72.92	0.753	0.772	0.846	0.591
Motif+NPC	82.22	0.845	0.826	0.845	0.801	50.00	0.610	0.571	0.615	0.364
CGI+N-gram+NPC	95.00	0.978	0.950	0.956	0.945	72.92	0.794	0.772	0.846	0.591
CGI+Motif+N-gram	94.45	0.989	0.944	0.945	0.945	72.92	0.760	0.772	0.846	0.591
CGI+Motif+NPC	82.22	0.849	0.826	0.845	0.801	50.00	0.617	0.571	0.615	0.364
Motif+N-gram+NPC	94.44	0.986	0.945	0.956	0.934	72.92	0.758	0.772	0.846	0.591
CGI+Motif+N-gram+NPC	94.44	0.986	0.945	0.956	0.934	72.92	0.763	0.772	0.846	0.591
Pattern of Feature	Cross-Validation					Independent-Testing				
	*Acc(%)*	*AUC*	*F*_*1*_	*Sn*	*Sp*	*Acc(%)*	*AUC*	*F*_*1*_	*Sn*	*Sp*
N-gram	88.89	0.969	0.890	0.901	0.878	70.83	0.823	0.781	0.962	0.409
NPC	57.78	0.600	0.537	0.489	0.666	52.08	0.502	0.303	0.192	0.909
CGI	49.44	0.494	0.326	0.244	0.745	58.33	0.615	0.375	0.231	1.000
Motif	79.45	0.795	0.798	0.812	0.779	64.58	0.638	0.691	0.731	0.545
CGI+N-gram	88.89	0.967	0.890	0.901	0.878	70.83	0.841	0.781	0.962	0.409
CGI+NPC	57.78	0.598	0.537	0.489	0.666	52.08	0.526	0.303	0.192	0.909
CGI+Motif	79.45	0.796	0.798	0.812	0.779	64.58	0.696	0.691	0.731	0.545
N-gram+NPC	88.33	0.972	0.884	0.890	0.878	79.17	0.806	0.828	0.923	0.636
Motif+N-gram	87.78	0.975	0.872	0.834	0.922	77.08	0.841	0.814	0.923	0.591
Motif+NPC	77.78	0.825	0.775	0.767	0.790	64.58	0.631	0.691	0.731	0.545
CGI+N-gram+NPC	88.33	0.972	0.884	0.890	0.878	79.17	0.813	0.828	0.923	0.636
CGI+Motif+N-gram	87.78	0.974	0.872	0.834	0.922	77.08	0.851	0.814	0.923	0.591
CGI+Motif+NPC	77.78	0.823	0.775	0.767	0.790	64.58	0.644	0.691	0.731	0.545
Motif+N-gram+NPC	88.33	0.978	0.879	0.846	0.922	77.08	0.830	0.814	0.923	0.591
CGI+Motif+N-gram+NPC	88.89	0.977	0.885	0.857	0.922	77.08	0.832	0.814	0.923	0.591

### Model selection

We determined the optimal models from training subset 1 and subset 2 by second layer model combination and then chose the final model by comparing the accuracy of the cross-validation on both models. However, we found for the subset 1 model that the cross-validation value for Acc was 94.45% and the independent-testing value was 72.92% on Acc.

For the subset 1 model, differences of performance between cross-validation and independent-testing on Acc and AUC were 21.53% and 0.229, respectively; for the subset 2 model, cross-validation of the subset 2 model for Acc was 88.33% lower than that of the subset 1 model, and differences of performance were 9.16% on Acc and 0.166 on AUC. From the above described, the subset 1 model has higher performance in learning, however, it worked not well in testing. In addition, the subset 1 model might have an overfitting phenomenon in the first layer because Motif encoding that could affect the performance of the second layer; overfitting of the subset 1 model (or any model) would engender poor prediction performance for data other than its own training data. To verify this issue, we used another training data from subset 2 as the testing data to evaluate the subset 1 model, and vice versa. In addition, we also used the training data from the building model as the testing data to evaluate the training quality of the model.

Evaluation results indicated that self-consistency compared to cross-validation increased 0.55% for the Acc indicator in subset 1. However, Acc increased by 1.67% in subset 2, indicating that the training quality of the model credible. Subset 1 was 6.12% higher than subset 2 in cross-validation and 5.00% higher in self-consistency. In contrast, subset 2 was 2.23% higher than subset 1 in exchange-testing, indicating that subset 2 was not only fault tolerant but also accurate with respect to prediction (Table E in S1 Supplement). So, we designed a formula (see Eq 14), it can calculate which training model who has the greater quality. Additionally, applying the formula, subset 2 was identified as the best-fit model for our system, because the score of the subset 2 model was higher than the score for the subset 1 model.

### Performance evaluation: distance between 35S enhancer and TLS of the gene

We found a correlation between gene activation by the 35S enhancer and the distance from the 35S enhancer to the TLS of the gene, indicating that the distance factor has an important significance (Fig 1A). We further analyzed the predictive performance of the EAT-Rice using different distance ranges and compared the predictive accuracy in training and independent-testing data. In addition, we compared the difference in predictive accuracy of EAT-Rice and TRIM platforms using different distance intervals.

**Fig 4:**
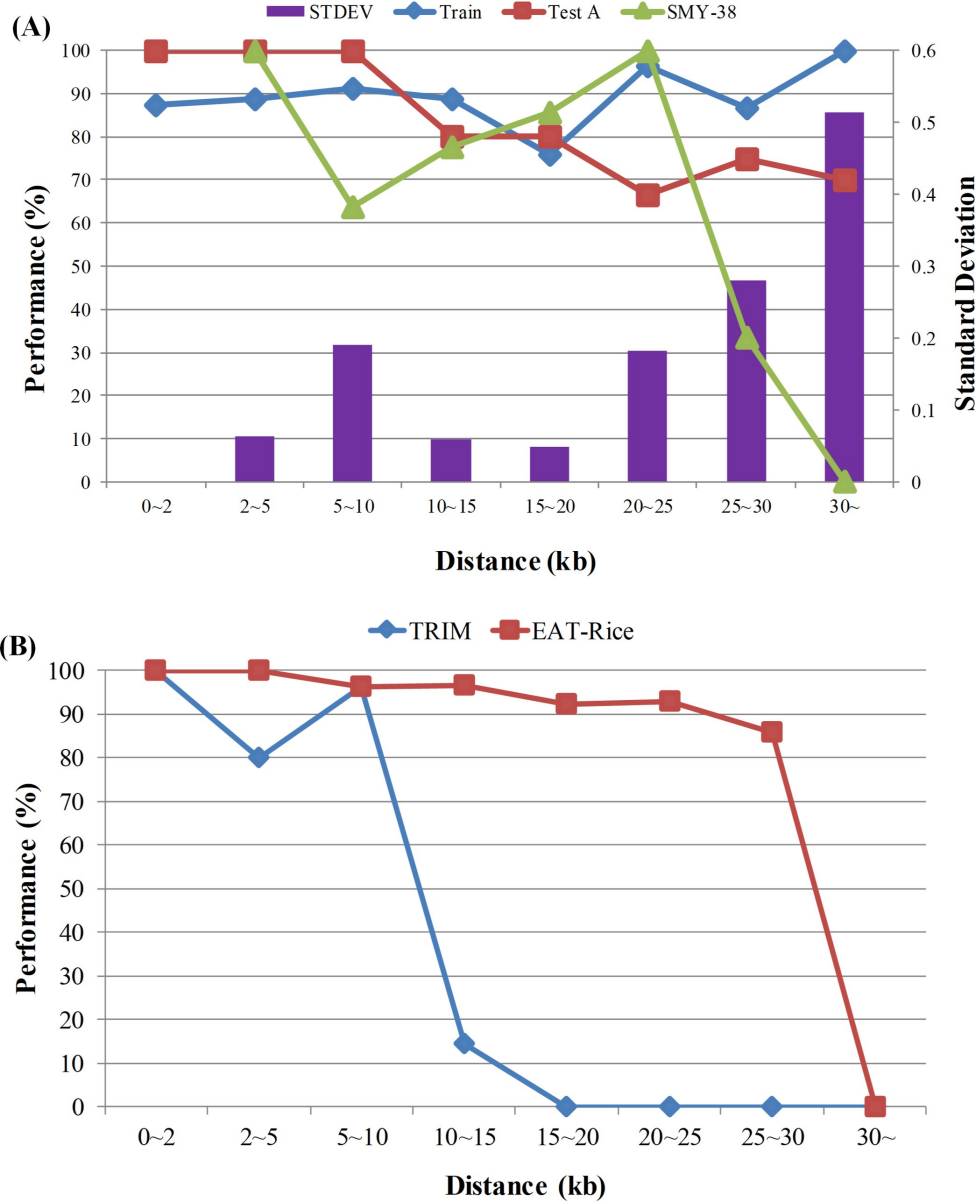
Performance evaluation in different distance ranges. (A) Assessment of EAT-Rice in different datasets. The value of Train, Test A and Test B are corresponding to left Y axis. Train indicates 5-fold cross-validation of training model. Test A indicates the performance of model with the original independent testing data. Test B indicates the performance of model with the new testing data collected after the EAT-Rice had been constructed. STDEV (cross line histogram) is the standard deviation of these three kinds of values, Train, Test A and Test B, and the value of STDEV is corresponding to the right Y axis (STDEV is non-available in the 0–2 range). (B) Assessment between EAT-Rice and TRIM. Y axis is the performance of accuracy.

First, we grouped training data of subset 2 and 48 independent-testing data based on different distance ranges and analyzed the predictive performance of EAT-Rice (Fig 4A; Table F in S1 Supplement). Among genes at >20 kb distances, Acc of training data showed an increasing trend, but independent-testing on Acc showed a decreasing trend. With the increase in the length of the DISTANCE sequence, the features generated by N-gram+NPC, the final model used for the EAT-Rice, were more consistent with sequence-specificity related to DISTANCE sequence, resulting in the observation of the overfitting phenomenon in EAT-Rice for gene over a 20 kb distance.

On the other hand, the T-DNA mutant lines were obtained from TRIM, however, we found that the states of flanking gene activation we identified were different from the database. To compare the performance of EAT-Rice and TRIM, we collected and analyzed 100 activated genes not used in subset 2 from 190 positive data in the training dataset. Performances of TRIM and EAT-Rice were 39.00% and 94.00% on Acc, respectively, applying this analysis (Data not shown). TRIM had reliable predictive accuracy when the gene distance was less than 10 kb, but less reliable predictive ability over 10 kb. For EAT-Rice, the performance gradually decreased, but predictive accuracy was eliminated for ranges beyond 30 kb (Fig 4B; Table G in S1 Supplement), indicating that the reliable predictive range of TRIM was approximately 10 kb up- and downstream of the T-DNA insertion site, however, EAT-Rice could predict more accurately than TRIM at greater gene distances. Overall, EAT-Rice out-performed TRIM with respect to the predictive accuracy of gene activation but due to overfitting, the predictive ability of EAT-Rice was reduced at distances of more than 20 kb.

## Discussion

In previous studies, we thought the enhancer has no bearing on activated genes when the orientation, location, or distance is different [17, 37, 45, 46]. However, our statistical results showed the distance factor may influence the probability of gene activation by the enhancer (Fig 1A). We speculated three reasons might causing the difference. 1) Previous investigations discussed the activation of this target genes by an endogenous enhancer, but the exotic 35S enhancer could cause nonspecific gene constitutive expression in the research. 2) The activation of a single enhancer was the focus of prior work; in contrast, our research objective focused on different insertion sites of enhancer from many mutant lines. Comparing the intention of the past research with ours are very different in this issue. 3) Finally, mammalian systems have been the target in previous work whereas ours is plants; the mechanisms of enhancers would be expected to be distinctive.

Previous work showed that the distance was a key factor to target gene influenced by the 35S enhancer on T-DNA activation-tagging [36]. The enhancer works only at a suitable distance and if the distance between the target gene and the enhancer is too far or too close, the enhancer-promoter interaction will be diminished [47, 48]. A similar mechanism exists in transgenic plants, where the interaction strength depends on the intensity of the enhancer and the sensitivity of the target gene promoter, and thus determines whether the distance barrier can be overcome [49, 50]. Although the sequence distance of suitable interaction for the 35S enhancer is unknown, prior work showed the impact of the 35S enhancer could be observed at a 78 kb distance [51].

In the first layer model, we captured three sequence fragments based on probable mechanisms of the enhancer and discovered that the rank of performance was MIDDLE > UPS1K > DISTANCE in the model built from N-gram (Table 2). We speculated the reason for DISTANCE sequence having the lowest accuracy was that it depended on the distance from the T-DNA insertion site to TLS of gene. The difference in the distance from insertion site to gene led to a varied sequence length (100 bp-30000 bp). The sequences may contain, for example, a gene coding region, promoter, or intergenic region. These sequences would produce excessive noise which would hinder classification, and led us to choose the important sequence by T-test.

It is noteworthy that the accuracy of MIDDLE is better than that of UPS1K. In general, we reasoned the performance of transcription was improved mainly by the enhancer interaction with promoter. We expected that the UPS1K sequence offered a critical message to augment the efficiency of classification and thought the sequence of DISTANCE and MIDDLE would provide less value. However, the result was not as expected. To check whether the MIDDLE could offer a useful message, we obtained randomly 180 fragments that were 301 bps from the rice chromosome to replace the original MIDDLE sequence. At the same time, to avoid taking repeat sequences, like retrotransposon elements or centromere region, we used BLAST method to compare the sequences of *Oryza* Repeat Database v3.3 offered by TIGR to randomly obtain sequences [52]. Then, we built the model by N-gram encoding with these sequences. Using independent-testing, the performance of model decreased by 18.75% of Acc, and then AUC decreased by 0.246 (Table H in S1 Supplement). The results illustrated that MIDDLE had a quite pronounced effect with regard to gene activation of 35S enhancer, suggesting the nearby relationship between the MIDDLE region sequence and enhancer.

The result of the first layer revealed the accuracy rank of the four features was N-gram > Motif > NPC > CGI. However, the result of the second layer showed the accuracy of the N-gram+Motif combination was less than the accuracy of N-gram alone. Although the principles of N-gram and Motif are similar, both are searching for specific fragments on sequences to encode, there are several differences between N-gram and Motif. N-gram used a 3–6 bp fragment from the random combination of nucleotides to encode, so its fragment may have no known biological significance. Motif collected cis-regulatory sequence fragments that have known biological significance in the plant kingdom. We anticipated N-gram and Motif to complement each other to enhance classification performance, however, the results demonstrated N-gram was a marked improvement over other combination. Perhaps, N-gram considered all nucleotide combinations, while Motif only considered data that was already confirmed by experiment. In the plant kingdom, the regulatory elements already confirmed are finite, and N-gram may substitute for the Motif function. Since the ND gene cannot confirm whether it is affected by 35S enhancer, it is deleted ND gene when data processing in this work.

Furthermore, we also participated the deleted 30 ND gene in training dataset, and following system structure to build the same model (Table I in S1 Supplement). The result indicated that the gene sequences of ND phenotype might include certain biological features of activated gene and produced an incorrect classification in the model.

## Conclusion

DNA sequence analysis and machine learning were used to build a two-layer model system. The system predicts the flanking gene expression activated by the 35S enhancer in rice mutant lines of T-DNA insertion activation-tagging. To avoid deviation caused by single machine learning, the two-layer model was implemented with LIBSVM algorithm in the first layer and NaiveBayesUpdateable algorithm in the second layer. The distance factor from the 35S enhancer to the translation start site of target gene is consider, so the possibility of target gene activation is estimated by logistic regression. Then, the feature weighting of the first layer model is based on the value of logistic regression. We retrieved three region sequences, including UPS1K, DISTANCE, and MIDDLE, and use these features including N-gram and NPC to encode. The accuracy of cross-validation is 88.33%, and the accuracy of independent-testing is 79.17%. When EAT-Rice is compared to TRIM, the accuracy of EAT-Rice is 55.00% greater than TRIM, and the confidence interval is in the range of 2–5 and 10–20 kb. We found a negative correlation between the distance on the genomic sequence and gene activation by the enhancer, for example, if the gene is closer to the enhancer, gene activation is more likely. For UPS1K, DISTANCE, and MIDDLE, the models constructed from MIDDLE and UPS1K contribute more to classified prediction, but the information offered from MIDDLE provided a greater contribution than UPS1K to the system for identifying activated gene in the model, suggesting the sequence context of MIDDLE may cause proteins to bind to the region and influence the interaction between the 35S enhancer and target gene. Finally, we have developed a system that predicts flanking gene expression activated by the CaMV 35S enhancer in T-DNA insertion activation-tagged rice mutants. We expect our system (EAT-Rice) can assist rice gene scientists in enhancing the efficiency of selecting candidate genes.

## Supporting information

S1 Supplement Supporting tables are provided in the attached document S1 Supplement. (PDF)

S1 Dataset The dataset is provided in the attached document S1 Dataset. (XLSX)

S1 Fig Proportional optimization of positive data and negative data of training dataset. The P/N ratio indicates the ratio of positive to negative data. Error bar is one-fold standard deviation (n = 3). MCC: Matthews Correlation Coefficient.
(PDF)

S2 Fig Schematic diagram for training activated genes grouping to Subset 1 and Subset 2. Ac-TFGs: the activated T-DNA flanking genes. The activated genes grouped by score which is stored in corresponding file.
(PDF)

S3 Fig Illustration of ATGCTA for significant pattern selection by T-test. The pattern alignment had done with ATGCTA in Ac and NAc group, respectively. Moreover, counting the match number for each gene and applying two sample T-test to analyze the significance of ATGCTA by the list of match number fetched from Ac and NAc group.
(PDF)
