# Supplementary material for: EAT-Rice: A predictive model for flanking gene expression of T-DNA insertion activation-tagged rice mutants by machine learning approaches
Source: PLoS Comput Biol. 2019 May 8;15(5):e1006942. doi: 10.1371/journal.pcbi.1006942 (PMC6505892; doi:10.1371/journal.pcbi.1006942)
Supplement: S1 Supplement — (PDF) [file pcbi.1006942.s001.pdf]

[*Supplement*] EAT-Rice: a predictive model for flanking gene expression of T-DNA insertion activation-tagged rice mutants by machine learning approaches

Chi-Chou Liao[1¶], Liang-Jwu Chen[1,2¶], Shuen-Fang Lo[3,4], Chi-Wei Chen[5,#a] and Yen-Wei Chu[1,3,6,7,8,#a*]
[1] Institute of Molecular Biology, National Chung Hsing University, Taichung 402, Taiwan
[2] Advanced Plant Biotechnology Center National Chung Hsing University, Taichung 402, Taiwan
[3] Agricultural Biotechnology Center, National Chung Hsing University, Taichung 402, Taiwan
[4] Institute of Molecular Biology, Academia Sinica, Taipei 115, Taiwan
[5] Department of Computer Science and Engineering, National Chung Hsing University, Taichung 402, Taiwan
[6] Biotechnology Center, National Chung Hsing University, Taichung 402, Taiwan.
[7] Ph.D. Program in Translational Medicine, National Chung Hsing University, Taichung 402, Taiwan.
[8] Rong Hsing Research Center For Translational Medicine, National Chung Hsing University, Taichung 402, Taiwan.
[#a] Institute of Genomics and Bioinformatics, National Chung Hsing University, Taichung 402, Taiwan

*Corresponding author: ywchu@nchu.edu.tw

[Tables]

All supplementary tables of this article mention are provided in here.

Table A. Using specific feature encoding for three kinds of sequence region.

**Sequence Region**	**Feature Encoding**			
	N-gram	Motif	NPC	CGI
Ups1k	●	●	●	●
Distance	●	○	●	○
Middle	●	○	●	○

The sequence region contains Ups1k, Distance and Middle and encoded by N-gram, Motif, NPC and CGI. Black circle indicates the sequence region with the feature encoding and white circle indicates the feature is not used in the corresponding sequence region.

Table B. Using multi-category machine learning algorithms to evaluate the second layer model.

Category	Algorithm	Cross-validation				Self-Consistency			
		Acc (%)	*AUC*	F_1	*AF*[a]	*Acc (%)*	*AUC*	F_1	*AF*
**Bayes**	BayesianLogisticRegression	90.833	0.908	0.910	0.909	95.000	0.950	0.950	0.950
	BayesNet	89.445	0.977	0.895	0.936	90.000	0.973	0.902	0.938
	NaiveBayes	50.000	0.500	0.000	0.250	50.000	0.500	0.000	0.250
	NaiveBayesSimple	92.222	0.981	0.921	0.951	93.611	0.977	0.935	0.956
	NaiveBayesUpdateable	92.222	0.982	0.921	0.952	93.611	0.977	0.935	0.956
**Functions**	LibLINEAR	90.556	0.906	0.907	0.907	93.889	0.939	0.939	0.939
	LibSVM	92.222	0.922	0.923	0.923	95.833	0.958	0.958	0.958
	LibSVM (grid)[b]	92.222	0.961	0.922	0.942	96.389	0.989	0.964	0.977
	Logistic	90.834	0.966	0.909	0.938	94.722	0.983	0.946	0.965
	MultilayerPerceptron	90.278	0.961	0.906	0.934	96.945	0.989	0.970	0.980
	RBFNetwork	88.056	0.952	0.880	0.916	93.334	0.975	0.933	0.954
	SimpleLogistic	91.389	0.937	0.915	0.926	93.334	0.960	0.933	0.947
	SMO	92.500	0.925	0.927	0.926	92.222	0.922	0.924	0.923
	VotedPerceptron	90.278	0.939	0.904	0.922	93.334	0.973	0.931	0.952
**lazy**	IB1	86.111	0.861	0.874	0.868	98.056	0.981	0.981	0.981
	IBk	90.833	0.946	0.910	0.928	98.334	0.999	0.983	0.991
	KStar	91.945	0.969	0.922	0.946	98.334	0.998	0.984	0.991
**meta**	AdaBoostM1	90.000	0.969	0.897	0.933	94.722	0.984	0.946	0.965
	AttributeSelectedClassifier	91.111	0.920	0.915	0.918	92.222	0.922	0.924	0.923
	Bagging	92.222	0.954	0.924	0.939	92.222	0.985	0.924	0.955
	ClassificationViaClustering	82.500	0.825	0.822	0.824	72.222	0.722	0.732	0.727
	ClassificationViaRegression	91.111	0.975	0.913	0.944	93.889	0.981	0.940	0.961
	CVParameterSelection	50.000	0.500	0.000	0.250	50.000	0.500	0.000	0.250
	Dagging	92.223	0.979	0.922	0.951	93.056	0.979	0.930	0.955
	Decorate	90.278	0.966	0.906	0.936	94.445	0.983	0.943	0.963
	END	90.556	0.903	0.909	0.906	94.167	0.956	0.940	0.948
	EnsembleSelection	92.222	0.971	0.924	0.948	92.222	0.984	0.924	0.954
	FilteredClassifier	91.944	0.922	0.921	0.922	92.222	0.922	0.924	0.923
	Grading	50.000	0.500	0.000	0.250	50.000	0.500	0.000	0.250
	LogitBoost	90.556	0.977	0.905	0.941	94.722	0.983	0.946	0.965
	MultiBoostAB	92.222	0.970	0.924	0.947	92.222	0.979	0.924	0.952
	MultiClassClassifier	90.834	0.966	0.909	0.938	94.722	0.983	0.946	0.965
	MultiScheme	50.000	0.500	0.000	0.250	50.000	0.500	0.000	0.250
	ClassBalancedND	90.556	0.903	0.909	0.906	94.167	0.956	0.940	0.948
	DataNearBalancedND	90.556	0.903	0.909	0.906	94.167	0.956	0.940	0.948
	nestedDichotomies.ND	90.556	0.903	0.909	0.906	94.167	0.956	0.940	0.948
	OrdinalClassClassifier	90.556	0.903	0.909	0.906	94.167	0.956	0.940	0.948
	RacedIncrementalLogitBoost	50.000	0.500	0.000	0.250	50.000	0.500	0.000	0.250
	RandomCommittee	90.278	0.941	0.903	0.922	98.334	0.999	0.983	0.991
	RandomSubSpace	90.556	0.977	0.905	0.941	91.944	0.977	0.921	0.949
	RotationForest	91.945	0.958	0.922	0.940	96.945	0.989	0.970	0.980
	Stacking	50.000	0.500	0.000	0.250	50.000	0.500	0.000	0.250
	StackingC	50.000	0.500	0.000	0.250	50.000	0.500	0.000	0.250
	ThresholdSelector	82.223	0.965	0.733	0.849	91.945	0.983	0.923	0.953
	Vote	50.000	0.500	0.000	0.250	50.000	0.500	0.000	0.250
**misc**	FLR	71.389	0.714	0.792	0.753	66.945	0.670	0.576	0.623
	HyperPipes	50.000	0.500	0.000	0.250	50.000	0.500	0.000	0.250
	VFI	91.111	0.977	0.913	0.945	91.667	0.973	0.918	0.946
**rules**	ConjunctiveRule	92.222	0.922	0.924	0.923	92.222	0.922	0.924	0.923
	DecisionTable	90.556	0.950	0.900	0.925	92.500	0.941	0.926	0.934
	DTNB	89.723	0.967	0.895	0.931	93.612	0.977	0.935	0.956
	JRip	91.389	0.922	0.913	0.918	92.778	0.930	0.930	0.930
	NNge	88.056	0.881	0.880	0.881	98.056	0.981	0.981	0.981
	OneR	92.222	0.922	0.924	0.923	92.222	0.922	0.924	0.923
	PART	88.611	0.918	0.882	0.900	95.000	0.981	0.950	0.966
	Ridor	89.723	0.898	0.897	0.898	93.334	0.934	0.932	0.933
	ZeroR	50.000	0.500	0.000	0.250	50.000	0.500	0.000	0.250
**trees**	ADTree	92.223	0.980	0.920	0.950	95.000	0.987	0.948	0.968
	BFTree	89.722	0.920	0.896	0.908	92.222	0.922	0.924	0.923
	DecisionStump	92.222	0.922	0.924	0.923	92.222	0.922	0.924	0.923
	FT	90.556	0.944	0.908	0.926	95.278	0.986	0.952	0.969

J48	90.556	0.903	0.909	0.906	94.167	0.956	0.940	0.948
J48graft	90.556	0.903	0.909	0.906	94.167	0.956	0.940	0.948
LADTree	92.223	0.969	0.921	0.945	95.556	0.990	0.955	0.973
LMT	91.944	0.937	0.921	0.929	93.334	0.960	0.933	0.947
NBTree	90.000	0.941	0.901	0.921	93.334	0.957	0.932	0.945
RandomForest	89.722	0.959	0.898	0.929	98.334	0.999	0.984	0.992
RandomTree	88.056	0.880	0.882	0.881	98.334	0.999	0.983	0.991
REPTree	91.945	0.919	0.919	0.919	92.500	0.933	0.926	0.930
SimpleCart	91.389	0.927	0.916	0.922	92.222	0.922	0.924	0.923

These seven categories including 69 algorithms were used to construct the second layer model, and the evaluation mechanism of cross-validation and self-consistency were used.

[a] AF represents the average of AUC and F_1.

[b] LibSVM (grid) represents that parameters of LibSVM are optimized for cost (C) and gamma (γ).

Table C. The analysis of distribution of gene number with four enhancer-associated properties.

Property	Gene number of Ac	Gene number of NAc	Total gene number
**Distance between 35S Enhancer and TLS of Gene (kb)**			
0~2	14	3	17
2~5	16	5	21
5~10	46	12	58
10~15	60	23	83
15~20	36	24	60
20~25	24	17	41
25~30	14	11	25
30~	6	17	23
**Gene Orientation**			
Positive	116	58	174
Negative	100	54	154
**Orientation of T-DNA Insertion**			
Positive	112	60	172
Negative	104	52	156
**Location of T-DNA insertion**			
Up-stream	107	59	166
Down-stream	104	52	156
Intragenic	5	1	6

Positive and Negative means sense and antisense strand. Up-stream or Down-stream means T-DNA inserts into upstream or downstream of target gene. Intragenic indicates T-DNA inserts into functional annotation of target gene.

Table D. The relationship between four enhancer-associated properties and gene activation by logistic regression.

Factor	Estimate Std.	Error	Z-value	P-value
(Intercept)[a]	1.448e+00	4.678e-01	3.094	0.00197
Distance[b]	-7.099e-05	1.430e-05	-4.963	6.93e-07
Gene Ori.	4.934e-02	2.461e-01	0.200	0.84112
Insertion Ori.	1.356e-01	2.474e-01	0.552	0.52116
T-DNA Location	1.700e-01	2.358e-01	0.721	0.47084

[a] Intercept indicates linear regression constant.

[b] The distance indicates from the 35S enhancer on T-DNA to translation starting site of gene.

Table E. Performance evaluation of models using Self-Consistency and Exchange-Testing.

Model	Self-Consistency[a]					Exchange-Testing[b]				
	Acc(%)	*AUC*	F_1	*Sn*	*Sp*	*Acc(%)*	*AUC*	F_1	*Sn*	*Sp*
CGI+Motif+N-gram	95.00	0.987	0.950	0.956	0.944	94.44	0.991	0.941	0.889	1.000
N-gram+NPC	90.00	0.965	0.902	0.922	0.878	96.67	0.998	0.966	0.933	1.000

CGI+Motif+N-gram is the model built by training subset 1. N-gram+NPC is the model built by subset 2.

[a] Self-Consistency means the prediction of itself training data as testing data.

[b] Exchange-Testing means the prediction of another training data as testing data.

Table F. System evaluation using testing data from different distance-based groups.

Distance (kb)	Train performance							
	Acc (%)	F_1	*Sn*	*Sp*	*Tp*	*Fn*	*Tn*	*Fp*
0 ~ 2	87.50	0.909	1.000	0.667	5	0	2	1
2 ~ 5	88.89	0.889	1.000	0.800	4	0	4	1
5 ~ 10	91.18	0.933	0.955	0.833	21	1	10	2
10 ~ 15	88.89	0.906	0.923	0.842	24	2	16	3
15 ~ 20	75.76	0.667	0.667	0.810	8	4	17	4
20 ~ 25	96.30	0.960	0.923	1.000	12	1	14	0
25 ~ 30	86.67	0.833	0.714	1.000	5	2	8	0
30 ~	100.00	1.000	1.000	1.000	1	0	8	0
**Distance (kb)**	**Test A performance**							
	Acc (%)	F_1	*Sn*	*Sp*	*Tp*	*Fn*	*Tn*	*Fp*
0 ~ 2	100.00	1.000	1.000	N.A.[a]	2	0	0	0
2 ~ 5	100.00	1.000	1.000	N.A.	2	0	0	0
5 ~ 10	100.00	1.000	1.000	N.A.	4	0	0	0
10 ~ 15	80.00	0.857	1.000	0.500	6	0	2	2
15 ~ 20	80.00	0.875	1.000	0.333	7	0	1	2
20 ~ 25	66.67	0.750	1.000	0.333	3	0	1	2
25 ~ 30	75.00	0.000	0.000	1.000	0	1	3	0
30 ~	70.00	0.000	0.000	0.778	0	1	7	2
**Distance (kb)**	**Test B performance**							
	Acc (%)	F_1	*Sn*	*Sp*	*Tp*	*Fn*	*Tn*	*Fp*
0 ~ 2	N.A.	N.A.	N.A.	N.A.	0	0	0	0
2 ~ 5	100.00	1.00	1.000	N.A.	1	0	0	0
5 ~ 10	63.64	0.778	0.875	0.000	7	1	0	3
10 ~ 15	77.78	0.857	0.857	0.500	6	1	1	1
15 ~ 20	85.71	0.923	1.000	0.000	6	0	0	1
20 ~ 25	100.00	1.000	1.000	1.000	3	0	3	0
25 ~ 30	33.33	0.500	0.333	N.A.	1	2	0	0
30 ~	0.00	0.000	0.000	N.A.	0	1	0	0

Train indicates 5-fold cross-validation in training model. Test A indicates independent-testing data for evaluating model. Test B indicates other testing data not included in this study.

[a] Data not available.

Table G. Predicting performance of EAT-Rice and TRIM for different distance-based groups.

Distance (kb)	TRIM			EAT-Rice		
	Acc (%)	*Tp*	*Fn*	*Acc (%)*	*Tp*	*Fn*
0 ~ 2	100.00	6	0	100.00	6	0
2 ~ 5	80.00	4	1	100.00	5	0
5 ~ 10	96.15	25	1	96.15	25	1
10 ~ 15	14.29	4	24	96.43	27	1
15 ~ 20	0.00	0	13	92.31	12	1
20 ~ 25	0.00	0	14	92.86	13	1
25 ~ 30	0.00	0	7	85.71	6	1
30 ~	0.00	0	1	0.00	0	1

Table H. Comparison of the performance of N-gram model using Middle region and random fragment sequence.

Model	Cross-Validation					Independent-Testing				
	Acc (%)	*AUC*	F_1	*Sn*	*Sp*	*Acc (%)*	*AUC*	F_1	*Sn*	*Sp*
Middle[a]	92.22	0.960	0.924	0.939	0.906	71.88	0.819	0.777	0.904	0.501
Rand[b]	96.39	0.989	0.964	0.950	0.978	53.13	0.573	0.685	0.943	0.046

[a] Middle means the model built by Middle sequences.

[b] Rand means the model built by rice sequence randomly captured form 12 rice chromosome.

Table I. Comparison of removal ND genes for the prediction performance of two models.

Model	Cross-Validation					Independent-Testing				
	Acc(%)	*AUC*	F_1	*Sn*	*Sp*	*Acc(%)*	*AUC*	F_1	*Sn*	*Sp*
Data358[a]	84.11	0.900	0.839	0.906	0.786	66.15	0.743	0.656	0.808	0.564
Data328[b]	88.33	0.972	0.884	0.890	0.878	79.17	0.806	0.828	0.923	0.636

[a] Data358 means the model without removing ND gene.

[b] Data328 means the model that remove ND gene
