# Supplementary material for: EAT-Rice: A predictive model for flanking gene expression of T-DNA insertion activation-tagged rice mutants by machine learning approaches
Source: PLoS Comput Biol. 2019 May 8;15(5):e1006942. doi: 10.1371/journal.pcbi.1006942 (PMC6505892; doi:10.1371/journal.pcbi.1006942)
Supplement: S1 Fig — The P/N ratio indicates the ratio of positive to negative data. Error bar is one-fold standard deviation (n = 3). MCC: Matthews Correlation Coefficient. (PDF) [file pcbi.1006942.s003.pdf]

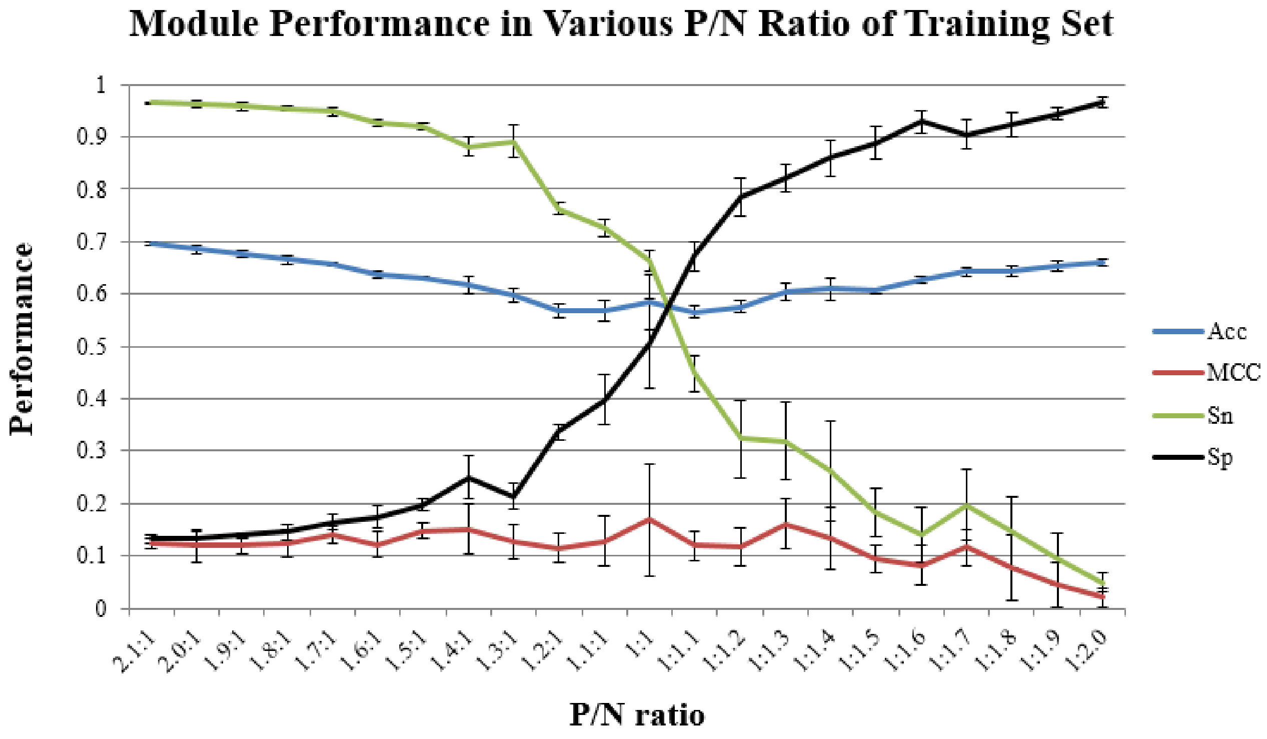

S1 Fig. Proportional optimization of positive data and negative data of training dataset. The P/N ratio indicates the ratio of positive to negative data. Error bar is one-fold standard deviation (n = 3). MCC: Matthews Correlation Coefficient.
