# Supplementary material for: EAT-Rice: A predictive model for flanking gene expression of T-DNA insertion activation-tagged rice mutants by machine learning approaches
Source: PLoS Comput Biol. 2019 May 8;15(5):e1006942. doi: 10.1371/journal.pcbi.1006942 (PMC6505892; doi:10.1371/journal.pcbi.1006942)
Supplement: S2 Fig — Ac-TFGs: the activated T-DNA flanking genes. The activated genes grouped by score which is stored in corresponding file. (PDF) [file pcbi.1006942.s004.pdf]

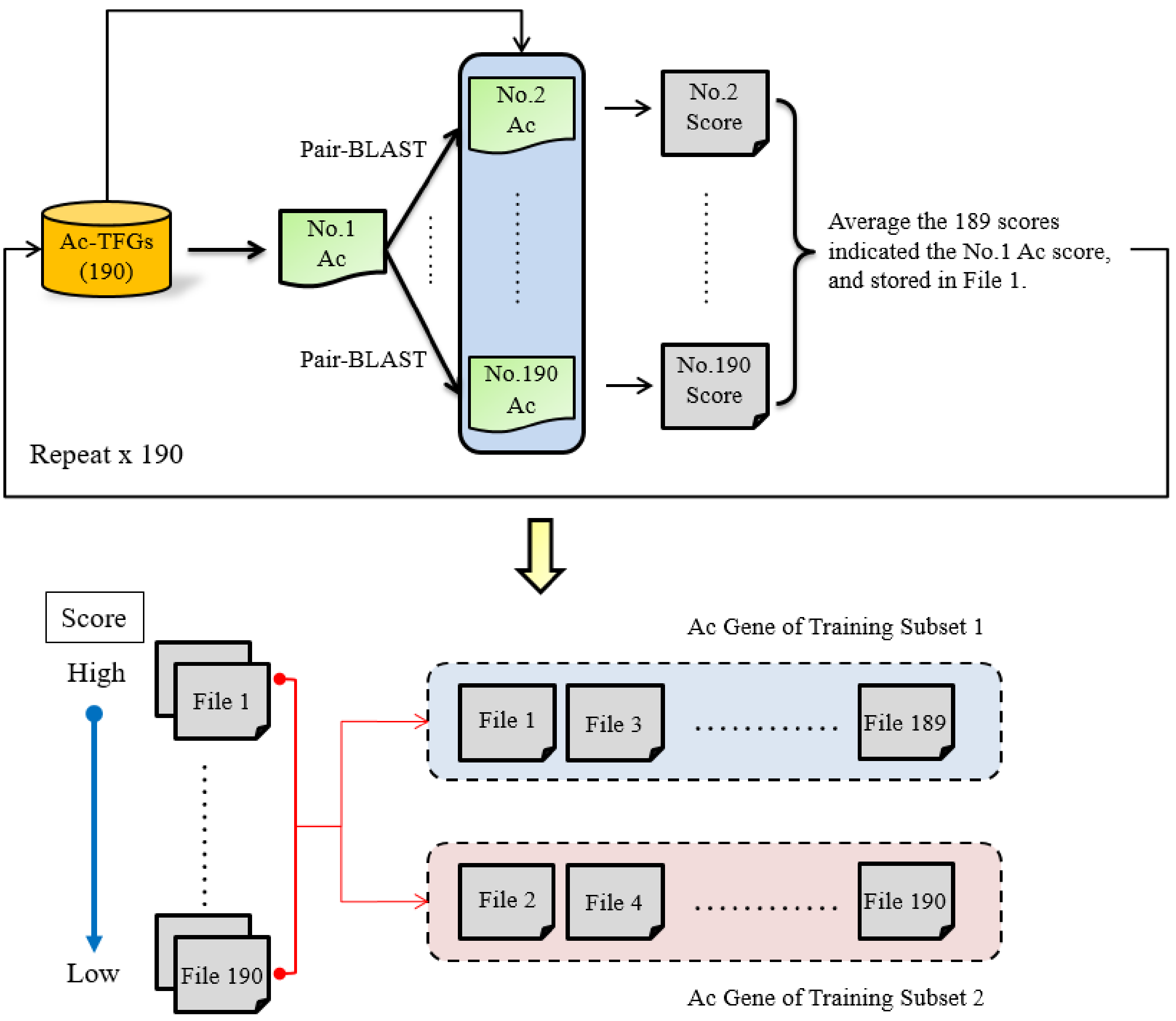

S2 Fig. Schematic diagram for training activated genes grouping to Subset 1 and Subset 2. Ac-TFGs: the activated T-DNA flanking genes. The activated genes grouped by score which is stored in corresponding file.
