# Supplementary material for: EAT-Rice: A predictive model for flanking gene expression of T-DNA insertion activation-tagged rice mutants by machine learning approaches
Source: PLoS Comput Biol. 2019 May 8;15(5):e1006942. doi: 10.1371/journal.pcbi.1006942 (PMC6505892; doi:10.1371/journal.pcbi.1006942)
Supplement: S3 Fig — The pattern alignment had done with ATGCTA in Ac and NAc group, respectively. Moreover, counting the match number for each gene and applying two sample T-test to analyze the significance of ATGCTA by the list of match number fetched from Ac and NAc group. (PDF) [file pcbi.1006942.s005.pdf]

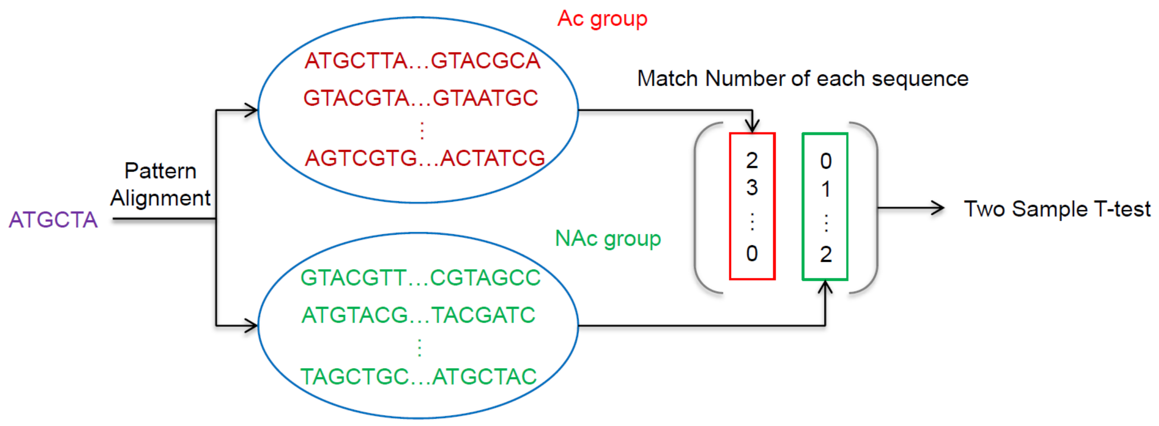

S3 Fig. Illustration of ATGCTA for significant pattern selection by T-test. The pattern alignment is done with ATGCTA in Ac and NAc group, respectively. Moreover, counting the match number for each gene and applying two sample T-test to analyze the significance of ATGCTA by the list of match number fetched from Ac and NAc group.
